# Bacteria-derived DNA in serum extracellular vesicles as a biomarker for gastric cancer

**DOI:** 10.1007/s00262-025-04175-0

**Published:** 2025-10-24

**Authors:** Kaoru Fujikawa, Takuro Saito, Atsunari Kawashima, Kentaro Jingushi, Daisuke Motooka, Shigeto Nakai, Takaomi Hagi, Kota Momose, Kotaro Yamashita, Koji Tanaka, Tomoki Makino, Tsuyoshi Takahashi, Yukinori Kurokawa, Kazutake Tsujikawa, Hisashi Wada, Hidetoshi Eguchi, Yuichiro Doki

**Affiliations:** 1 Department of Gastroenterological Surgery, Graduate School of Medicine, The University of Osaka, Osaka, Japan; 2 Department of Clinical Research in Tumor Immunology, Graduate School of Medicine, The University of Osaka, Osaka, Japan; 3 Department of Urology, Graduate School of Medicine, The University of Osaka, Osaka, Japan; 4 Laboratory of Stem Cell Regeneration and Adaptation, Graduate School of Pharmaceutical Sciences, The University of Osaka, Osaka, Japan; 5 Compound Library Screening Center, Graduate School of Pharmaceutical Sciences, Department of Infection Metagenomics, The University of Osaka, Osaka, Japan; 6 Center for Supporting Drug Discovery and Life Science Research, The University of Osaka, Osaka, Japan

**Keywords:** 16S rRNA gene, Bacterial DNA, Extracellular vesicles, Gastric cancer, Tumor-infiltrating lymphocyte, T cell exhaustion

## Abstract

Bacterial flora is present in various parts of the human body, and bacterial 16S rRNA genes have been detected in the bloodstream. Distinct blood microbiomes have been identified in various diseases, including cancer, and are thought to play a role in disease pathogenesis. In this study, we conducted a 16S rRNA metagenomic analysis of serum extracellular vesicles from 89 patients with gastric cancer (GC) and 25 healthy donors. We identified lower levels of *Bacteroidetes* and *Actinobacteria* and higher levels of *Firmicutes* in patients with GC than in healthy donors. By integrating this characteristic bacterial DNA profile, we developed a BAF index, defined as the ratio of *Bacteroidetes* and *Actinobacteria* to *Firmicutes,* which exhibited high sensitivity for detecting GC in both the discovery and validation cohorts, suggesting its potential utility as a screening tool. A high BAF index was significantly associated with an advanced tumor stage and poor prognosis. Moreover, a high BAF index was linked to an immunosuppressive tumor microenvironment, which may contribute to the unfavorable outcomes observed in these patients. These findings indicate that circulating bacterial signatures may serve as promising biomarkers for GC.

**Supplementary Information:**

The online version contains supplementary material available at 10.1007/s00262-025-04175-0.

## Introduction

Gastric cancer (GC) accounts for approximately 10% of all malignancies worldwide [1]. As early-stage GC often lacks clinical symptoms, there is an urgent need to establish a biomarker that can easily and accurately diagnose GC. Although carcinoembryonic antigen (CEA) and cancer antigen 19–9 (CA19-9) are commonly used tumor markers, their positivity rates in early-stage GC are below 10%, making them insufficient for screening purposes [2, 3]. Consequently, the National Comprehensive Cancer Network (NCCN) guidelines do not recommend their use for diagnostic screening [4]. Therefore, the development of a simple, sensitive, and noninvasive screening test for the early detection of GC is imperative.

Bacteria are present within tumors and influence tumorigenesis and antitumor immune responses [5–9]. For example, *Lactobacillus reuteri* within melanoma tumors promotes interferon-γ production by CD8^+^ T cells via the secretion of indole-3-aldehyde [8]. In pancreatic cancer, the tumor-specific microbiome has been shown to suppress tumor growth by activating M1 macrophages and CD8^+^ T cells following microbial ablation [5]. *Helicobacter pylori* is a well-established carcinogen [10–12] that modulates the tumor immune microenvironment by inducing regulatory T cells (Tregs), thereby contributing to the immune suppressive environment [13, 14]. These findings suggest that diverse bacterial species influence cancer development and tumor immunity.

Extracellular vesicles (EVs) are secreted by both prokaryotic and eukaryotic cells and can carry bacterial DNA (b-DNA) when secreted by bacteria [15, 16]. Although bacteria were once believed to be absent from the bloodstream under normal conditions, studies have shown that circulating EVs can contain bacterial components, including b-DNA [17–19]. In urothelial carcinoma, a higher proportion of *Firmicutes* DNA in serum EVs correlates with reduced tumor-infiltrating T cells, decreased T cell activation, and poorer prognosis in patients treated with anti-programmed cell death protein 1 (PD-1) therapy [20]. Similarly, tumor-specific b-DNA signatures have been detected in serum EVs from patients with renal cell carcinoma and shown to distinguish patients from healthy donors (HDs) [21]. These findings suggest that GC may also have a distinct b-DNA profile in serum EVs, which could serve as a potential diagnostic biomarker for GC.

In this study, we evaluated the diagnostic utility of the b-DNA profile in serum EVs for GC. Specifically, we aimed to develop a minimally invasive method for early-stage GC detection and identify potential bacterial biomarkers present in the serum EVs of patients with GC.

## Materials and methods

### Patients’ recruitment and data

This study included patients with histologically proven pStage I–III GC who underwent curative resection between 2015 and 2023 at Osaka University Hospital, Japan. Clinicopathological factors were obtained from medical records. Tumors were staged according to the Japanese classification of gastric carcinoma [22]. Histological type classification was based on the Lauren classification [23]. Relapse-free survival (RFS) was defined as the time from the operation to either disease progression or death from any cause. Overall survival (OS) was defined as the time from the operation to death from any cause. Written informed consent was obtained from each patient in accordance with the Declaration of Helsinki.

### Comparison of bacterial information in serum EVs

**Fig. 1:**
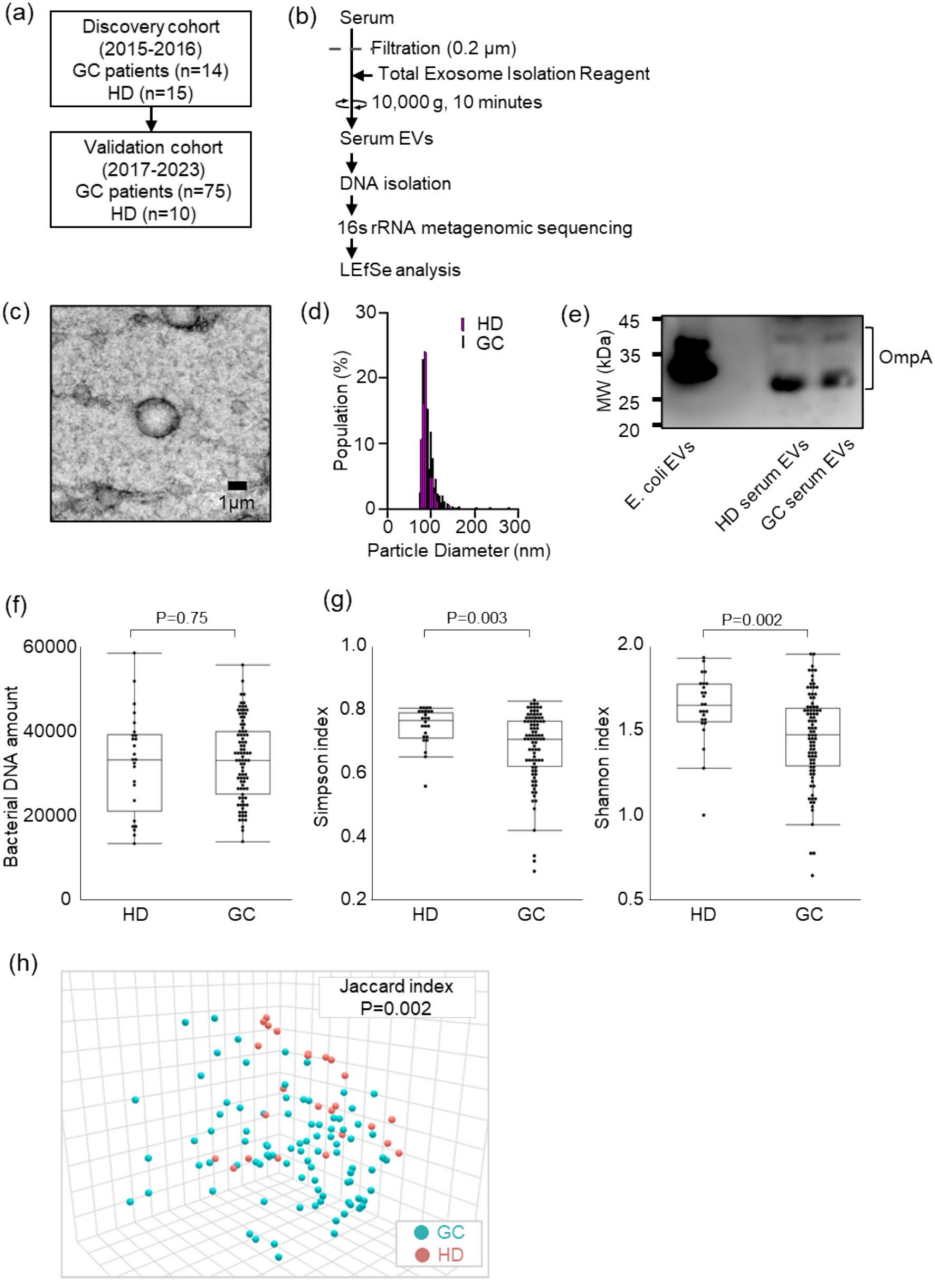
Extracellular vesicle (EV) collection from serum and isolation of bacteria-derived DNA (b-DNA). **a** Study flow diagram. **b** Methods for processing and analyzing serum samples. **c**, **d** Representative results of transmission electron microscopic **c** and nanoparticle **d** analysis of EVs isolated from serum samples. Black bar indicates 1 µm. **e** Western blot analysis of serum EVs from a patient with gastric cancer (GC) and a healthy donor (HD) using an anti-OmpA antibody. **f** DNA amount in EVs in 100 μL of serum from 89 patients with GC and 25 HDs. Comparison between the two groups was performed using the Mann–Whitney U test. **G** Alpha diversity analysis (left: Simpson, right: Shannon) for serum EVs from 89 patients with GC and 25 HDs. Comparison between the two groups was performed using the Mann–Whitney U test. **h** Beta diversity plots for serum EVs from 89 patients with GC and 25 HDs

A flow diagram of the study is shown in Fig. 1a. Differences in b-DNA information in serum EVs were investigated between HDs and patients with GC in a discovery cohort (2015–2016), and the differences were confirmed in a validation cohort (2017–2023). HDs were defined as those without a current malignant disease or a medical history of cancer.

### Collection of EVs and isolation of bacteria-derived DNA

Serum EVs were collected and isolated as described previously (Fig. 1b) [20, 21]. Whole blood samples were collected in Venoject II tubes (TERUMO, Tokyo, Japan) immediately before surgery. Within 3 h after sample collection, all samples were centrifuged at 1200 × *g* for 15 min, and supernatants were stored at −80 ℃. No medications containing antibiotics or probiotics were routinely administered before surgery. For EV isolation, serum samples were centrifuged at 2000 × *g* for 30 min and filtered through a 0.2-μm syringe filter (KURABO, Osaka, Japan). Serum EVs were isolated using the Exosome Isolation Kit (serum) (Thermo Fisher Scientific, Waltham, MA, USA) according to the manufacturer’s protocol. EVs isolated from serum samples were confirmed using transmission electron microscopy (TEM), nanoparticle analysis, and western blotting for OmpA, a bacteria-derived EV marker (Fig. 1c–e). b-DNA from serum EVs was purified using the QIAamp® Circulating Nucleic Acid Kit (QIAGEN, Hilden, Germany) according to the manufacturer’s protocol.

### TEM analysis

TEM was performed according to a previously reported method [24]. EV samples were placed on a Formvar carbon-coated nickel grid for 1 h and fixed with 2% paraformaldehyde before observation with an HT7800 microscope (HITACHI, Tokyo, Japan).

### Nanoparticle measurement

The size and concentration of the EVs were measured using qNano Gold (Izon Science, Christchurch, New Zealand). Data were analyzed using the Izon Control Suite Software (V3.February 3, 2001).

### Western blotting analysis

EV samples were lysed with Laemmli sodium dodecyl sulfate sample buffer without 2-mercaptoethanol and separated on a 12% gel through sodium dodecyl sulfate–polyacrylamide gel electrophoresis, followed by transfer onto a polyvinylidene difluoride membrane using a Bio-Rad semidry transfer system (1 h, 12 V). The membranes were probed with anti-*Escherichia coli* OmpA Pab (1:1000) primary antibodies at 4 °C overnight. Membranes were incubated with horseradish peroxidase-conjugated secondary antibodies against mouse immunoglobulin (1:5000) for 1 h at room temperature. Chemiluminescence was detected using an Amersham Imager 680 (GE Healthcare). As OmpA varies in length among bacterial species, serum EV proteins were detected at different locations than in *E. coli* [25].

### 16S metagenomic sequencing

The PCR-amplified V1–V2 regions of the bacterial 16S ribosomal RNA gene were sequenced on a MiSeq platform (Illumina, San Diego, CA, USA). QIIME version 2.202002 was used to process the raw sequencing data. The Simpson and Shannon indices indicated population diversity in the samples. Linear discriminant analysis (LDA) effect size (LEfSe) was calculated using the Microbiome Analyst web platform, and only LDA scores ≥ 3.5 were listed as previously reported (Fig. 1b) [21].

### Multicolor flow cytometry

Tumor samples were collected from surgically dissected specimens. Fresh tumor tissues were minced and digested to a single-cell suspension using a Tumor Dissociation Kit for humans (Miltenyi Biotec, Germany) and a gentle MACS Dissociator (Miltenyi Biotec) according to the manufacturer’s instructions. The cell suspension was applied to a 70-µm nylon cell strainer (BD Biosciences, USA), and red blood cells were lysed using BD Pharm Lyse for 2 min. Dead cells and debris were removed through centrifugation in an isodensity Percoll solution (Pharmacia Biotech). Surface marker staining was performed after FcR blocking for 15 min using Human TruStain FcX Fc receptor blocking solution (BioLegend, USA). Cells were incubated with antibodies against surface antigens and fixable viability dye (eBioscience, USA) at 4 °C for 30 min. After incubation, cells were washed, fixed, and permeabilized with a fix/perm solution (BD Biosciences) at 4 °C for 15 min. Cells were then stained with antibodies against intracellular molecules at 4 °C for 30 min. Cells were analyzed on a BD LSRFortessa using the FACSDiva software (BD Biosciences). Details of the antibody clones used for staining the cell molecules are shown in Table S1. The representative staining patterns and gating strategies are shown in Fig. S1.

### Statistical analysis

Statistical analyses and visual quantification were performed using the JMP software (JMP 16.1, SAS Institute). Associations were assessed in case–case comparisons using the chi-square test for categorical variables and the Mann–Whitney U test for continuous variables. The diagnostic abilities were evaluated using the receiver operating characteristic (ROC) analysis. The formula for the BAF index was created using logistic regression analysis, as previously reported [21]. The Kaplan–Meier method was used to calculate survival rates, and log-rank tests were used to compare the two groups. Statistical significance was set at a two-sided *P*-value of < 0.05.

## Results

### Patient characteristics

The clinical characteristics of the patients with GC and HDs are presented in Table 1. The median age of patients with GC was 75 years, and 66.3% were men. Compared with HDs, patients with GC were older and had a higher percentage of men. The pathological stage of GC was I in 39 patients (43.8%), II in 24 patients (27.0%), and III in 26 patients (29.2%). *H. pylori* infection was detected in 44 patients (55.6%).

**Table 1:** Patient characteristics

		GC n = 89	HD n = 25	*P*–value
Age	Median [IQR]	75 [68–81]	63 [50–72]	< 0.001
Sex	Male / female	59 / 30	11 / 14	0.039
Location	U / M / L	26 / 28 / 35	–	
Lauren classification	Intestinal / diffuse	55 / 34	–	
pT status	1 / 2 / 3 / 4	39 / 18 / 17 / 4	–	
pN status	0 / 1 / 2 / 3	40 / 18 / 11 / 21	–	
pStage	1 / 2 / 3	39 / 24 / 26	–	
*H.pylori*	Negative / positive	35 / 44	–	

### Differences in bacterial information from serum EVs

EVs isolated from serum samples were confirmed using TEM, nanoparticle analysis, and western blotting for OmpA, a bacteria-derived EV marker (Fig. 1c–e). There were no significant differences in the total amount of bacterial DNA between patients with GC and HDs (Fig. 1f). Patients with GC showed significantly lower α-diversity, as assessed using the Simpson and Shannon indices, compared with HDs (Fig. 1g). In the principal coordinate analysis based on the Jaccard index, b-DNA profiles in serum EVs from patients with GC were significantly different from those in HDs (*P* = 0.002) (Fig. 1h).

### LEfSe analysis of b-DNA information in serum EVs from patients with GC and HDs

**Fig. 2:**
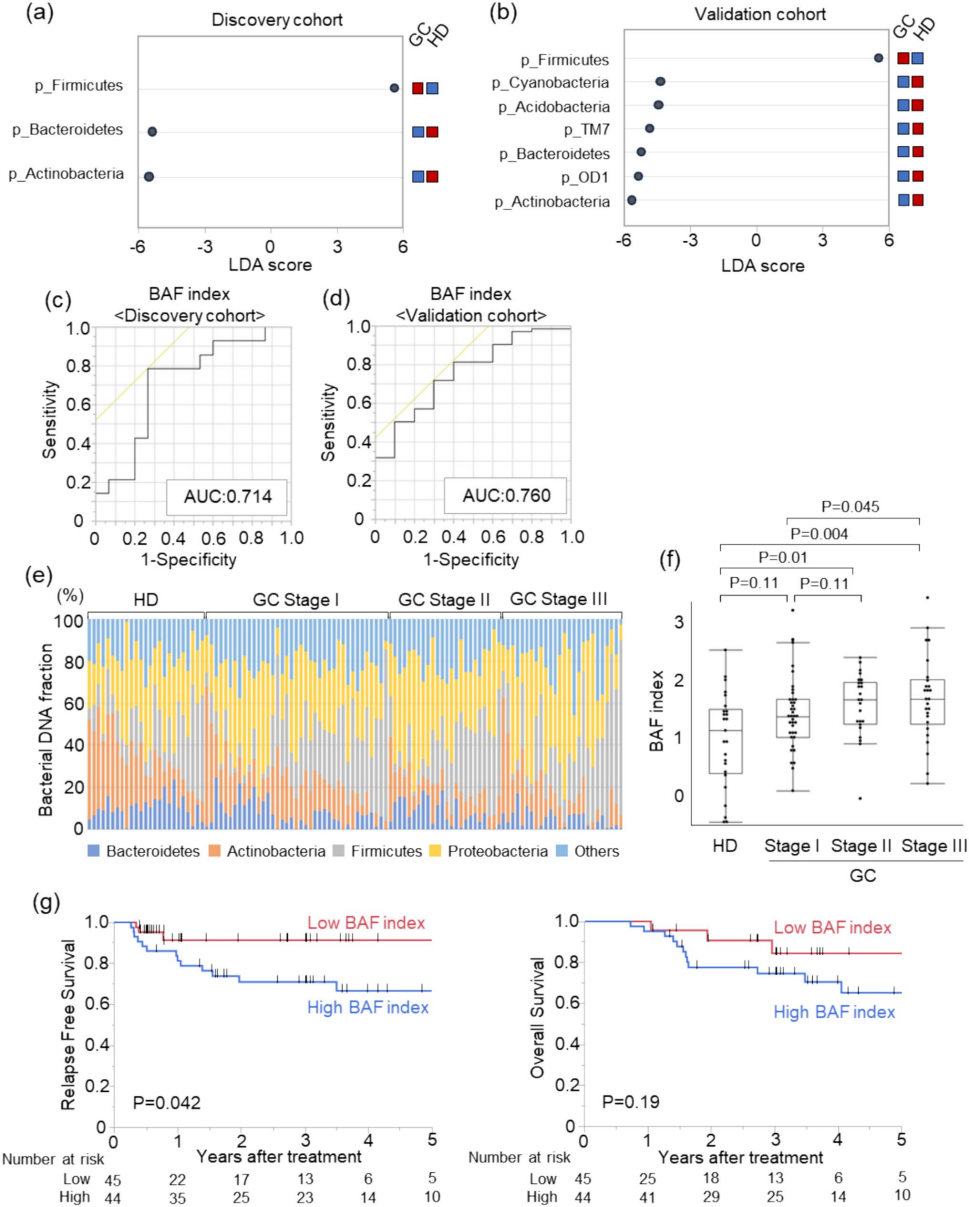
Comparison of the abundance of bacteria-derived DNA (b-DNA) in serum extracellular vesicles (EVs) between patients with gastric cancer (GC) and healthy donors (HDs). **a**, **b** Linear discriminant analysis effect size (LEfSe). Distinctive bacterial information detected in serum EVs from patients with GC and HDs in the discovery **a** and validation **b** cohorts. The biological classification classes are denoted by initials (p: phylum). **c**, **d** ROC curve showing the diagnostic performance of the BAF index in patients with GC in the discovery **c** and validation **d** cohorts. **e** Distribution of b-DNA in patients with HDs and GC stratified by pStage. **f** BAF index in HDs and patients with GC stratified by pStage. **g** Relapse-free survival (RFS) and overall survival (OS) after surgery in patients with GC and high and low BAF indices. Statistical analyses were performed using log-rank tests

The b-DNA profiles of serum EVs were compared between patients with GC (2015–2016; *n* = 14) and HDs (*n* = 15) as the discovery cohort using LEfSe analysis. Analysis at the bacterial phylum level (level 2) revealed that patients with GC exhibited higher LDA scores for *Firmicutes* and lower LDA scores for two operational taxonomic units (OTUs), *Bacteroidetes* and *Actinobacteria* (Fig. 2a). The proportion of *Bacteroidetes* was significantly higher and that of *Actinobacteria* tended to be higher in HDs than in patients with GC, whereas the proportion of *Firmicutes* tended to be higher in patients with GC than in HDs (Fig. S2a).

The results of the discovery cohort were confirmed in a validation cohort, where b-DNA information in serum EVs was compared between patients with GC (2017–2023; *n* = 75) and HDs (*n* = 10) using LEfSe analysis. Patients with GC had higher LDA scores for *Firmicutes* and lower LDA scores for six OTUs, including *Bacteroidetes* and *Actinobacteria* (Fig. 2b), indicating that the same OTUs were identified in both cohorts. These results were further supported by the relative abundance of each bacterial phylum. The proportion of *Bacteroidetes* tended to be higher and that of *Actinobacteria* was significantly higher in HDs than in patients with GC, whereas that of *Firmicutes* tended to be higher in patients with GC than in HDs (Fig. S2b).

### BAF index using b-DNA information showed high sensitivity for patients with GC

Next, we investigated whether the bacterial profiles of serum EVs could be used to distinguish patients with GC from HDs. A novel scoring system, the BAF index, was developed using the proportion of b-DNAs from *Bacteroidetes*, *Actinobacteria*, and *Firmicutes* and was calculated as follows: BAF index = 1.55732699032807 + (0.000073939795799846 × *Firmicutes*)—(0.0000087290440622304 × *Bacteroidetes*)—(0.00011995497036724 × *Actinobacteria*).

In the discovery cohort, the area under the curve (AUC) for diagnosing GC was 0.641 for *Bacteroidetes*, 0.630 for *Actinobacteria*, 0.624 for *Firmicutes*, and 0.714 for the BAF index (Fig. 2c, Fig. S3a). When the cutoff value for the BAF index was set to 1.604, which the ROC analysis indicated would optimize both sensitivity and specificity, the sensitivity was 78.6%, specificity was 73.3%, and accuracy was 75.9%.

In the validation cohort, the AUC for diagnosing GC was 0.760 for *Actinobacteria*, 0.621 for *Firmicutes*, and 0.760 for the BAF index (Fig. 2d, Fig. S3b). Applying the same cutoff value (1.604) from the discovery cohort, the sensitivity was 42.7%, specificity was 100.0%, and accuracy was 56.0%. The BAF index distinguished patients with pStage I GC from HDs, with an AUC value of 0.621, sensitivity of 33.3%, specificity of 80.0%, and accuracy of 51.6%. (Fig. S3c). Furthermore, a logistic regression analysis showed that the BAF index was an independent factor for GC diagnosis in all patients (odds ratio [OR] 4.40, 95% confidence interval [CI] 1.39–13.92, *P* = 0.012) as well as older age (Table 2).

**Table 2:** Univariate and multivariate analyses for GC diagnosis in the whole cohort

		Univariate		Multivariate	
		OR (95% CI)	*P*–value	OR (95% CI)	*P*–value
Age	< 65 ≥ 65	Reference 4.94 (1.91–12.8)	0.001	Reference 5.45 (1.88–15.8)	0.002
Sex	Female Male	Reference 2.50 (1.01–6.18)	0.047	Reference 1.92 (0.70–5.30)	0.21
BAF index	< 1.604 ≥ 1.604	Reference 3.57 (1.23–10.4)	0.019	Reference 4.40 (1.39–13.9)	0.012

*CI* = confidence interval, *OR* = odds ratio

We also compared the positivity of conventional tumor markers, CEA and CA19-9, with that of the BAF index in patients with GC by applying the same cutoff value (1.604) from the discovery cohort. The positivity was 33.7% for CEA, 15.7% for CA19-9, and 47.2% for the BAF index in patients with GC (Table 3). Accordingly, the diagnostic accuracy of the BAF index for detecting GC tended to be higher than that of CEA (*P* = 0.067) and was significantly higher than that of CA19-9 (*P* < 0.001). Among the patients with pStage I GC, the positivity was 28.2% with CEA, 10.3% with CA19-9, and 33.3% with the BAF index. The diagnostic accuracy of the BAF index for detecting pStage I GC did not differ significantly from that of CEA (*P* = 0.62) but was significantly higher than that of CA19-9 (*P* = 0.014). Given its higher sensitivity and diagnostic accuracy, the BAF index shows promise as a screening tool for GC.

**Table 3:** Positivity of markers in patients with gastric cancer and healthy donors

		GC_all stages (*n* = 89)	GC_Stage I (*n* = 39)	HDs (*n* = 25)
CEA	±	30 / 59 (34% / 66%)	11 / 28 (28% / 72%)	NE
CA19-9	±	14 / 75 (16% / 84%)	4 / 35 (10% / 90%)	NE
BAF index*	±	42 / 47 (47% / 53%)	13 / 26 (33% / 67%)	5 / 20 (20% / 80%)

^*^ The cutoff value for the BAF index was 1.604. Abbreviations: *NE*, Not evaluated

### Association of b-DNA profile in serum EVs with GC progression and prognosis

The proportions of b-DNAs in serum EVs from HDs and patients with GC, stratified by pStage, are shown in Fig. 2e. As the GC stage advanced, the relative abundance of *Bacteroidetes* and *Actinobacteria* gradually decreased, whereas that of *Firmicutes* increased. Accordingly, the BAF index increased in the advanced stages, showing significant differences between HDs and pStage II (*P* = 0.01) and pStage III (*P* = 0.004) and between pStage I and pStage III (*P* = 0.045) (Fig. 2f). When patients with GC were divided into two groups based on the median value of the BAF index value, RFS was significantly better, and OS tended to be better in patients with a low BAF index than in those with a high BAF index (5-year RFS rate: 91.3% vs. 66.5%; *P* = 0.042, 5-years OS rate: 84.2% vs. 64.9%; *P* = 0.19) (Fig. 2g, Table S2). Moreover, when the cutoff value of the BAF index was set at 1.522, which was determined in ROC analysis to optimize both sensitivity and specificity for predicting cancer recurrence, the sensitivity, specificity, and accuracy were 81.3%, 57.5%, and 61.8%, respectively (Fig. S4a), and the AUC was 0.682. Stratification of patients with GC based on this cutoff value revealed significantly higher RFS and a trend toward higher OS in patients with a low BAF index than in those with a high BAF index (5-year RFS rate: 91.3% vs. 66.5%; *P* = 0.044, 5-year OS rate: 84.2% vs. 64.9%; *P* = 0.19) (Fig. S4b).

### Association of b-DNA profile in serum EVs with intratumoral immune cell status

**Fig. 3:**
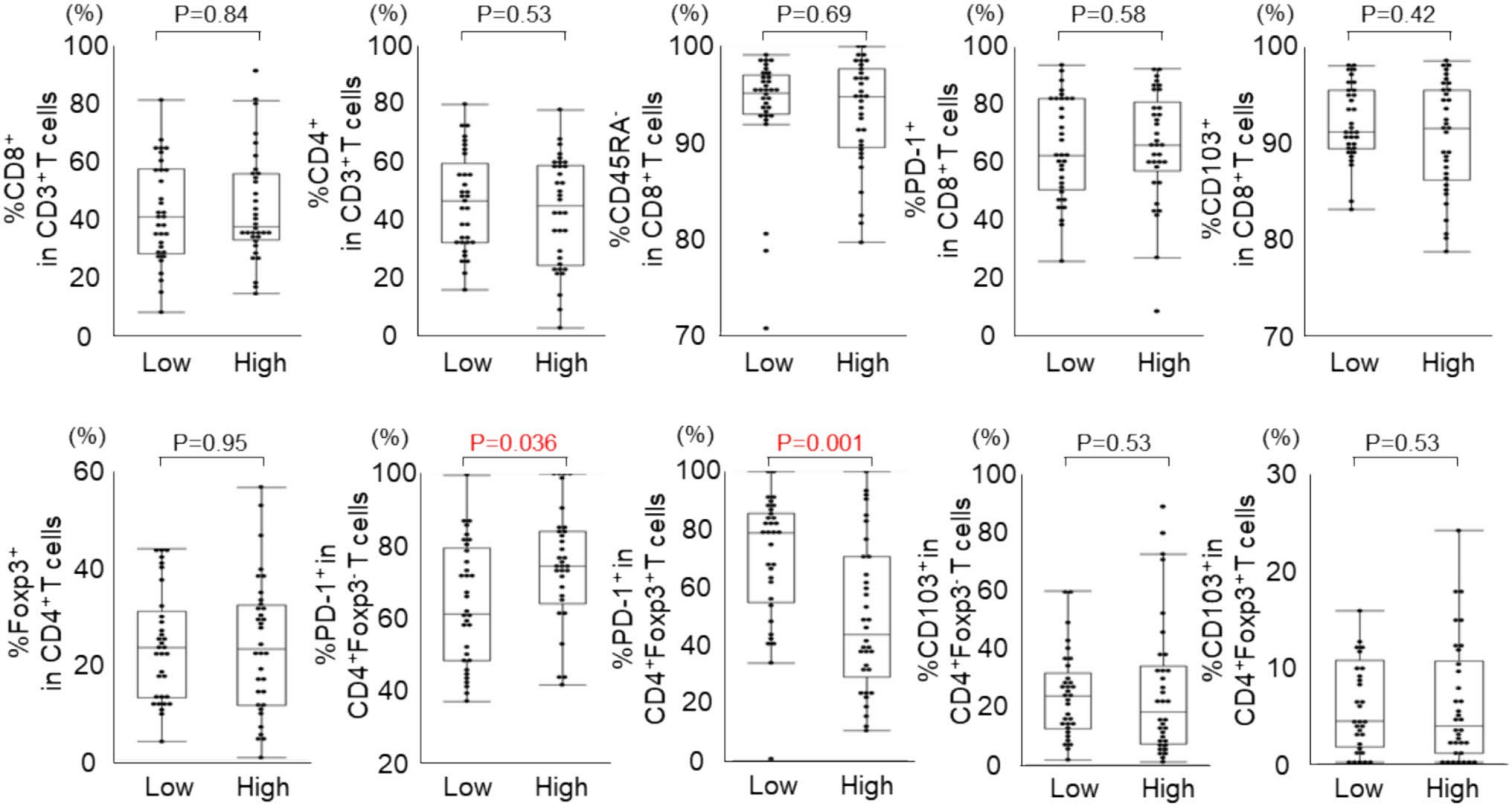
Correlation between bacteria-derived DNA (b-DNA) and tumor-infiltrating lymphocytes (TILs). Quantification of immune cell populations in GC TILs. The frequency of CD8^+^ in CD3^+^ T cells, CD4^+^ in CD3^+^ T cells, CD45RA^−^ in CD8^+^ T cells, PD-1^+^ in CD8^+^ T cells, CD103^+^ in CD8^+^ T cells, FoxP3^+^ in CD4^+^ T cells, PD-1^+^ in FoxP3^−^CD4^+^ T cells, PD-1^+^ in FoxP3^+^CD4^+^ T cells, CD103^+^ in FoxP3^−^CD4^+^ T cells, and CD103^+^ in FoxP3^+^CD4^+^ T cells were analyzed between high and low BAF index. Comparison between the two groups was performed using the Mann–Whitney U test

Among the 87 patients with GC, fresh tumor tissue samples were available for 67 patients, from which tumor-infiltrating lymphocytes (TILs) were isolated and analyzed using flow cytometry (Fig. S1). The frequencies of PD-1 and CD103 expression on CD8^+^ T cells, CD4^+^FoxP3^−^ T cells, and CD4^+^FoxP3^+^ regulatory T cells (Tregs) were assessed, and the relationship between b-DNA profiles and TIL characteristics was evaluated. PD-1 expression was significantly higher in CD4^+^FoxP3^−^ T cells and lower in Tregs of patients with high *Bacteroidetes* abundance, whereas the opposite trend was observed in patients with high *Firmicutes* abundance (Fig. S5). Furthermore, patients with a high BAF index also showed significantly increased frequency of PD-1 expression in CD4^+^FoxP3^−^ T cells and decreased frequency in Tregs (Fig. 3). As PD-1 expression in T cells generally indicates an exhausted status, these findings suggest that a high BAF index may be associated with an immunosuppressive tumor microenvironment, potentially contributing to unfavorable clinical outcomes.

## Discussion

This study demonstrated the clinical significance of bacterial profiles in serum EVs from patients with GC. The DNA composition in serum EVs from patients with GC differed markedly from that of HDs, showing consistently higher levels of *Firmicutes* and lower levels of *Bacteroidetes* and *Actinobacteria* in both the discovery and validation cohorts. Based on these three bacterial signatures, we developed a BAF index that enables the highly sensitive detection of GC. A high BAF index is significantly associated with advanced tumor stage and poor prognosis, potentially reflecting an immunosuppressive tumor microenvironment.

EVs are universal intercellular signaling vehicles found in both eukaryotes and bacterial systems [16]. Bacterial components have been identified in the human bloodstream [17, 18], and EVs containing these components have been reported to modulate systemic immune responses [26, 27]. Furthermore, we previously examined the relationship between b-DNA information in EVs and tumor immune status, focusing on its association with immunotherapy efficacy [20, 21]. In urothelial carcinoma, lower levels of *Firmicutes* in serum EVs are correlated with increased infiltration and activation of tumor-infiltrating T cells, resulting in an improved response to anti-PD-1 therapy [20]. Similarly, in renal cell carcinoma, lower levels of *Bacteroidia* in serum EVs were associated with a lower abundance of tumor-infiltrating Tregs, contributing to the enhanced therapeutic efficacy of anti-PD-1 therapy [21]. These findings suggest that specific b-DNA signatures in serum EVs may reflect the tumor immune status and predict the response to immunotherapy. Experimentally, EVs derived from *Cutibacterium acnes* have been shown to promote tumor growth in renal cell carcinoma both in vitro and in vivo, and *C. acnes* DNA has been detected in serum EVs of patients with renal cell carcinoma [28]. These results imply that EVs released by specific bacteria within tumor tissues may contribute to tumor progression. In this study, specific b-DNA signatures in serum EVs from patients with GC were associated with an immunosuppressive tumor microenvironment. However, the correlation with immunotherapy efficacy could not be evaluated because of the lack of data from patients treated with immunotherapy. Further investigation is warranted to determine whether b-DNA profiles in serum EVs from patients with GC are predictive of the response to immunotherapy.

In our previous study, b-DNA profiles from various samples, including serum EVs and stools, were analyzed using 16S RNA sequencing. These findings suggest that the bacterial content of serum EVs may reflect that of the gut microbiota [20]. Consistent with this, our results showed an increased proportion of *Firmicutes* and decreased proportions of *Bacteroidetes* and *Actinobacteria* in the b-DNA content of serum EVs. Previous studies on the gastric microbiota of patients with GC have reported enrichment of *Firmicutes* and a decrease in *Bacteroidetes* and *Actinobacteria* [29–31]. These findings support the possibility that EVs originate from the gastrointestinal tract and subsequently enter the bloodstream.

One of the main challenges in GC screening is the low sensitivity of conventional tumor markers. Reported sensitivity ranges from 16–68% for CEA and 14–68% for CA19-9 [2–4]. In our cohort, the BAF index demonstrated a higher sensitivity than CEA and CA19-9. For patients with pStage I GC, the sensitivity of the BAF index was better than that of the CEA and CA19-9. These results indicate that the BAF index offers superior diagnostic performance compared with conventional tumor markers, particularly in early-stage GC. Therefore, the BAF index may serve as a promising screening tool for the early detection of GC.

This study had some limitations. First, it was a retrospective study with a small and imbalanced cohort in terms of age and sex. Furthermore, the AUC for diagnosing GC was modest, and the ability to distinguish stage I GC from HD was limited. Large-scale, multicenter studies are required to validate the findings of the present study and improve performance. Second, detailed background information on HDs was not examined in this study. Comprehensive profiling of patients with HDs, including information on comorbidities, should be considered for a more accurate assessment of the screening performance of the BAF index.

In conclusion, the bacterial profile of serum EVs may enable minimally invasive and highly sensitive diagnosis of GC and prognostic prediction in patients with GC. Further large-scale validation studies are warranted to confirm these findings and explore their clinical utility.

## Supplementary Information

Below is the link to the electronic supplementary material.

Supplementary file1 ( 143 kb)

Supplementary file2 ( 540 kb)

## Data Availability

The datasets generated during and/or analyzed during the current study are available from the corresponding author on reasonable request.
