# Supplementary material for: Bacteria-derived DNA in serum extracellular vesicles as a biomarker for gastric cancer
Source: Cancer Immunol Immunother. 2025 Oct 24;74(11):346. doi: 10.1007/s00262-025-04175-0 (PMC12552193; doi:10.1007/s00262-025-04175-0)
Supplement: Supplementary file 1 — Supplementary file1 ( 143 kb) [file 262_2025_4175_MOESM1_ESM.pdf]

Supplementary Table 1. Key resources table

Reagent or Resource	Source
Chemicals	
DNase I	Nippon Gene
Total Exosome Isolation Reagent (from serum)	Thermo Fisher
Excel Band 3-color High Range Protein Marker	Cosmobio
Tumor Dissociation Kit for humans	Miltenyi Biotec
Isodensity Percoll solution	Pharmacia Biotech
Human TruStain FcX	BioLegend
Fixable viability dye	eBioscience
Fixation/Permeabilization Kit	BD Biosciences
Software and algorithms	
QIIME	Illumina
Microbiome Analyst	McGill University, Dr. Jianguo Xia's Lab
qNanoGold	Ion
HT7800	HITACHI
LSR Fortessa X20 flow cytometer	BD Biosciences
JMP	SAS Institute
Antidobies	
Anti-E.coli OmpA Pab	Antibody Research Corporation
Anti-Rabbit IgG HRP-linked Antibody	CST
Anti-Human CD45 (Clone:HI30)	BD Biosciences
Anti-Human CD3 (Clone:UCHT1)	BioLegend
Anti-Human CD8a (Clone:RPA-T8)	BioLegend
Anti-Human CD4 (Clone:OKT4)	BioLegend
Anti-Human Foxp3 (Clone:236A/E7)	Thermo Fisher
Anti-Human PD-1 (Clone:EH12.1)	BD Biosciences
Anti-Human CD103 (Clone:Ber-ACT8)	BioLegend

Supplementary Table 2. Patient characteristics based on the BAF index

		Low BAF index (n = 45)	High BAF index (n = 44)	P value
Age	Median [IQR]	76 [68–80]	73 [68–81]	0.76
Sex	Male / Female	33 / 12	26 / 18	0.15
Location	U / M, L	11 / 34	15 / 29	0.32
Lauren classification	Intestinal / Diffuse	31 / 14	24 / 20	0.16
pT status	1 / 2-4	21 / 24	18 / 26	0.58
pN status	0 / 1-3	24 / 21	16 / 28	0.11
pStage	1 / 2-3	25 / 20	14 / 30	0.023
H.Pylori	Negative/Positive	17 / 26	18 / 18	0.35
