# Supplementary material for: Bacteria-derived DNA in serum extracellular vesicles as a biomarker for gastric cancer
Source: Cancer Immunol Immunother. 2025 Oct 24;74(11):346. doi: 10.1007/s00262-025-04175-0 (PMC12552193; doi:10.1007/s00262-025-04175-0)
Supplement: Supplementary file 2 — Supplementary file2 ( 540 kb) [file 262_2025_4175_MOESM2_ESM.pdf]

Supplementary Figure 1

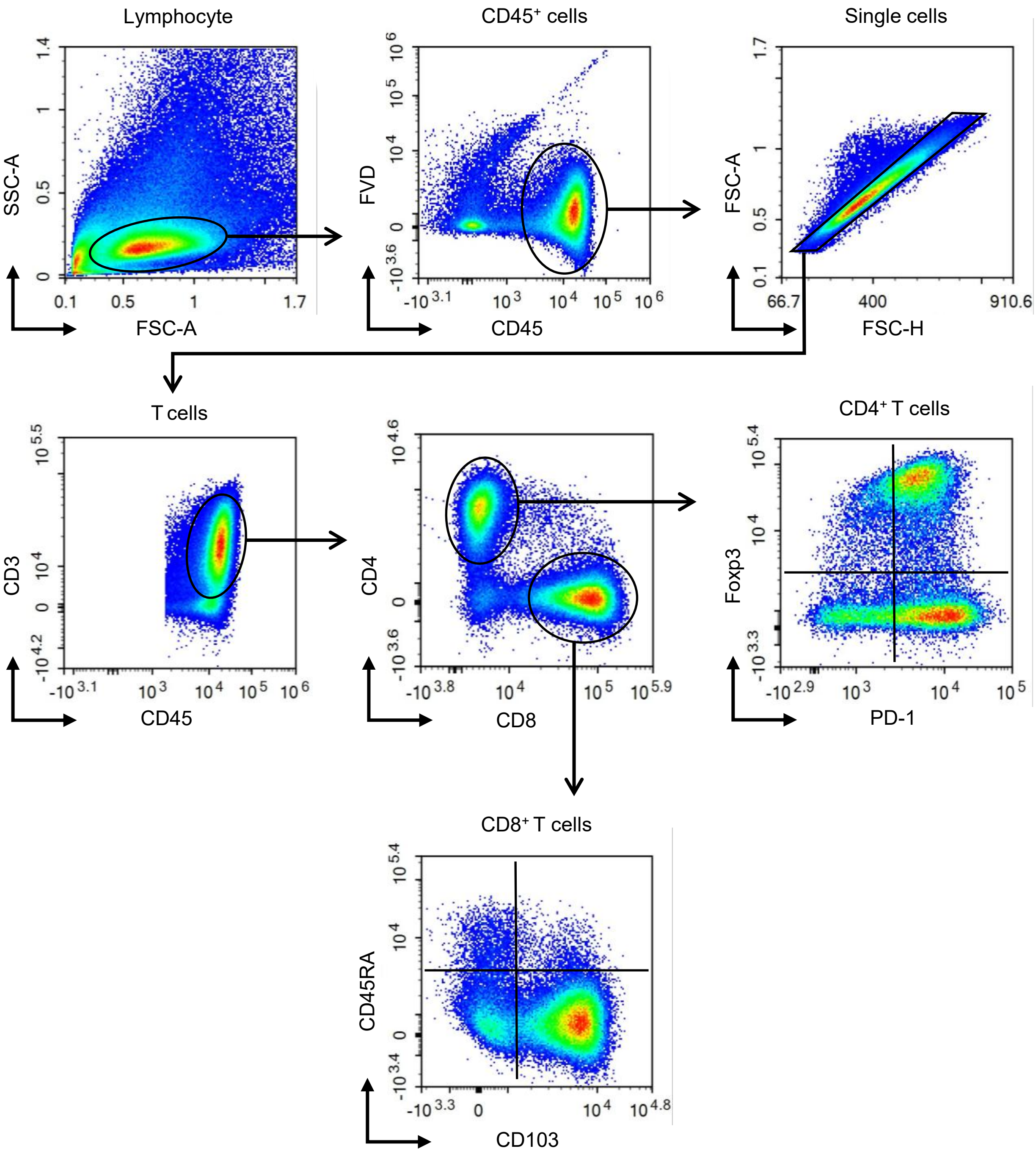

Supplementary Fig. S1 Representative flow cytometry images.

Supplementary Figure 2

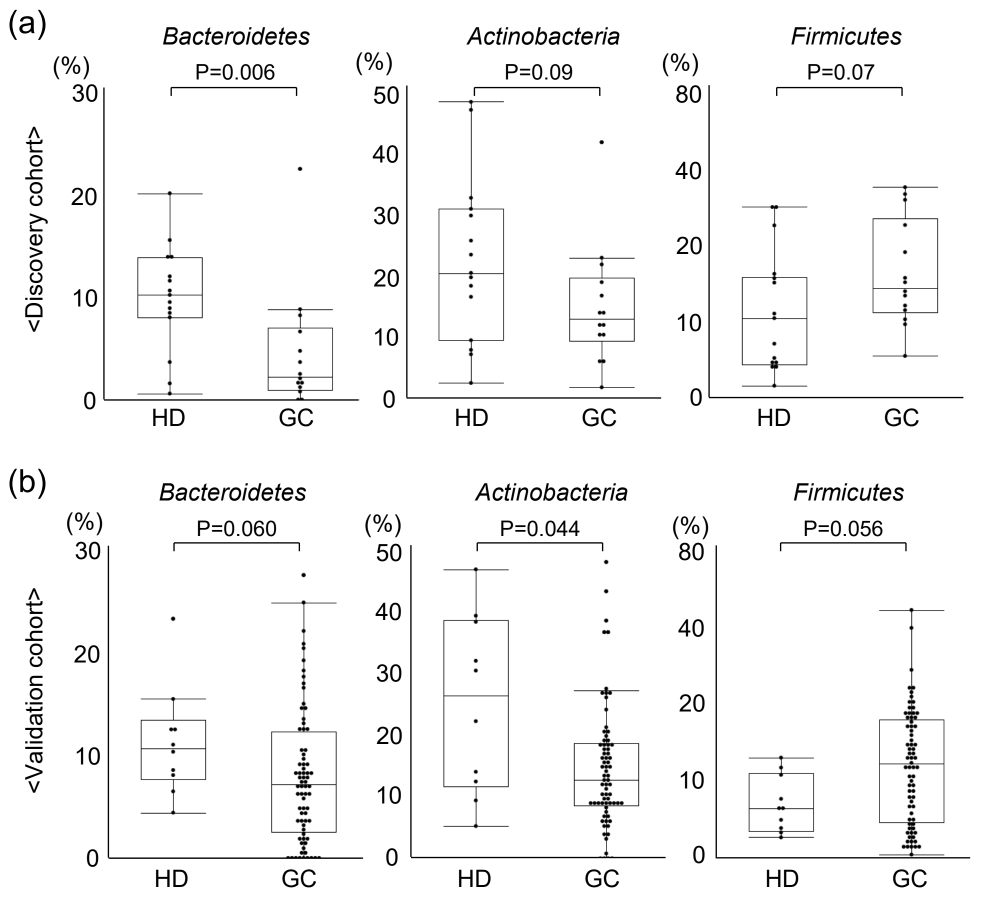

Supplementary Fig. S2 Comparison of the abundance of bacteria-derived DNA (b-DNA) in serum extracellular vesicles between patients with GC gastric cancer (GC) and healthy donors (HDs).

(a) Comparison of the abundance of *Bacteroidetes*, *Actinobacteria*, and *Firmicutes* between patients with GC and HDs in the discovery cohort. (b) Comparison of the abundance of *Bacteroidetes*, *Actinobacteria*, and *Firmicutes* between patients with GC and HDs in the cohort and validation cohorts. Comparison between the two groups was performed using the Mann–Whitney U test.

Supplementary Figure 3

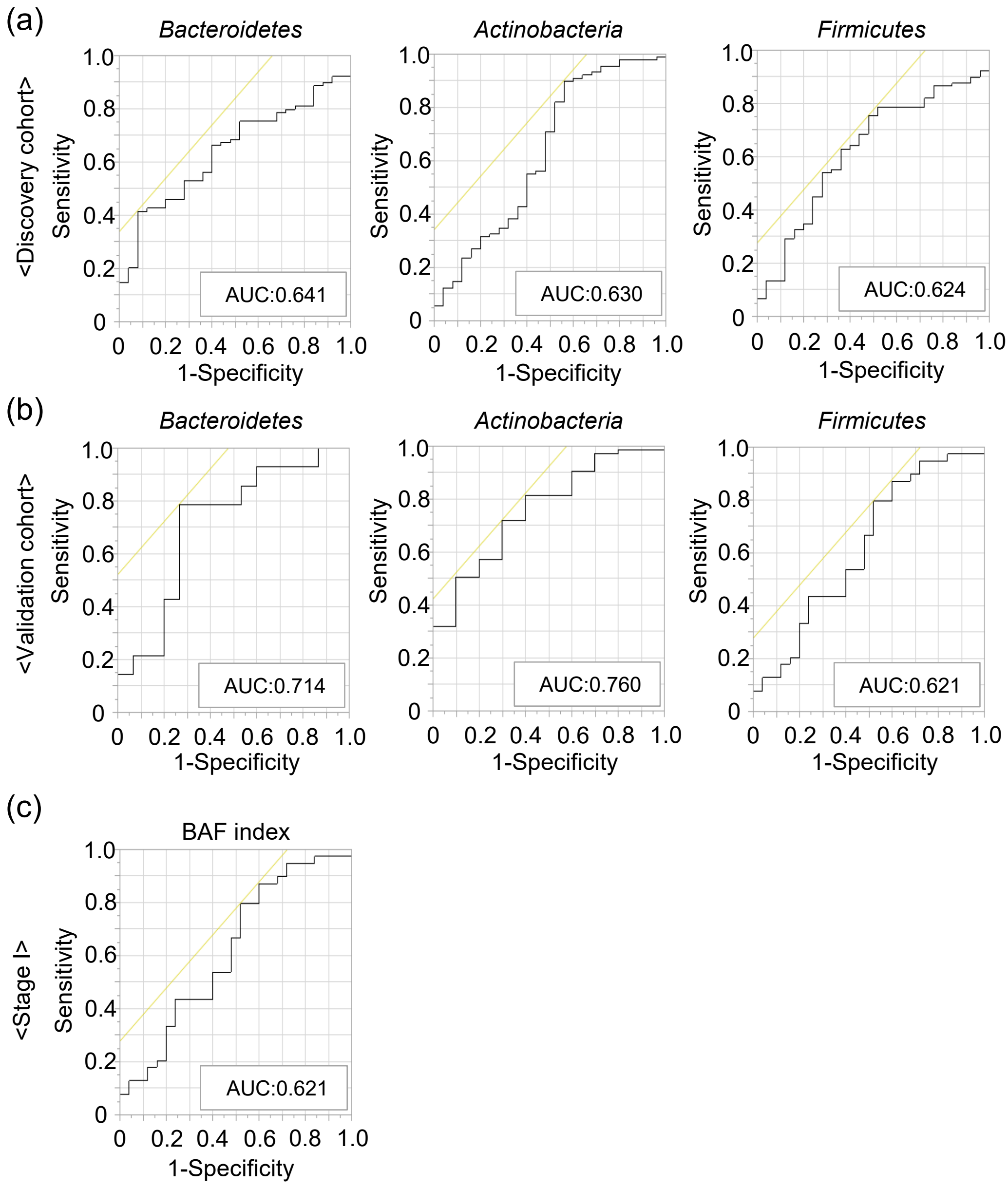

Supplementary Fig. S3 Receiver operating characteristic (ROC) curves for the diagnostic accuracy in patients with gastric cancer (GC).

(a) ROC curve showing diagnostic performance for *Bacteroidetes*, *Actinobacteria*, and *Firmicutes* in the discovery cohort. (b) ROC curve showing diagnostic performance for *Bacteroidetes*, *Actinobacteria*, and *Firmicutes* in the validation cohort. (c) ROC curve showing diagnostic performance of the BAF index in patients with GC with pStage I disease. AUC, area under the curve.

Supplementary Figure 4

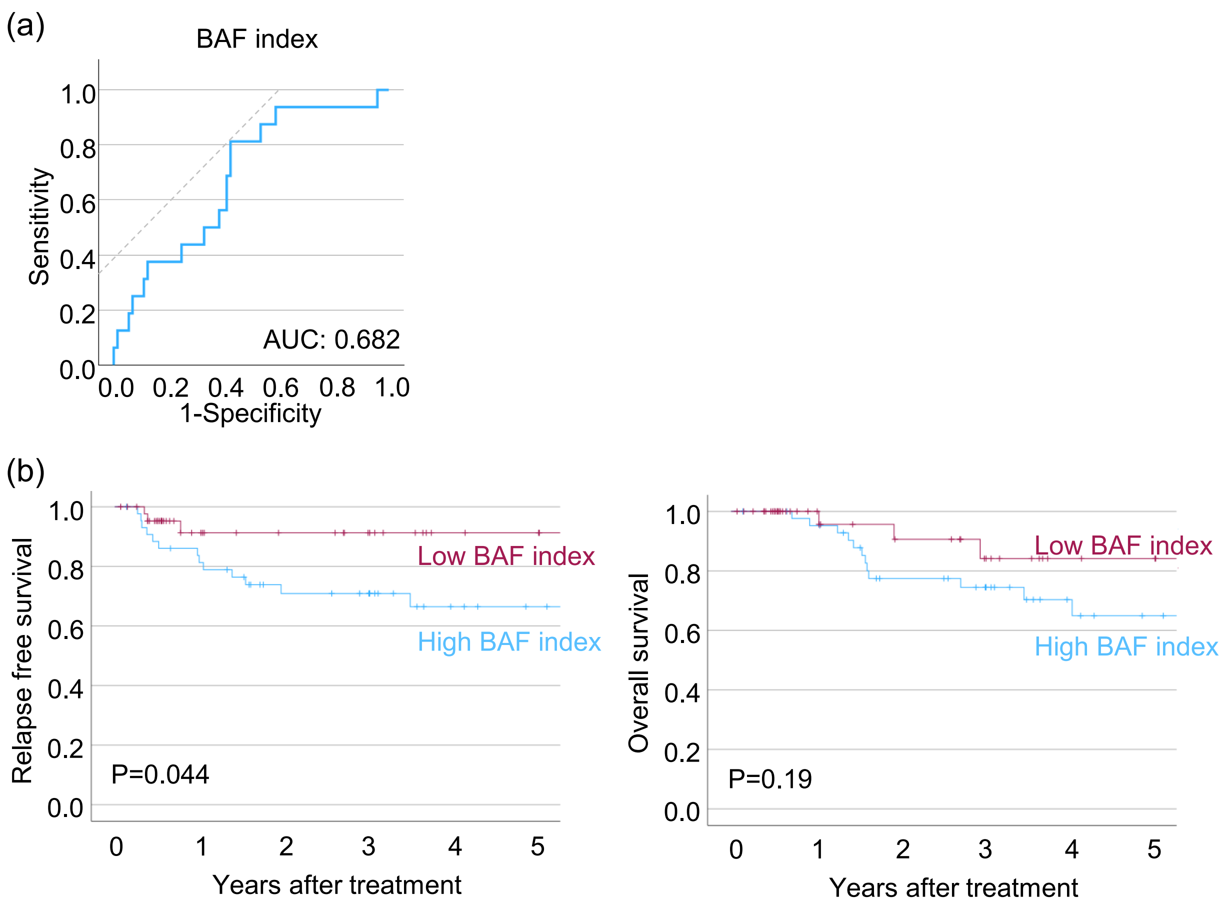

Supplementary Fig. S4 Correlation between BAF index and prognosis in patients with gastric cancer (GC).

(a) Receiver operating characteristic curve showing the prognostic performance of the BAF index in patients with GC. (b) Relapse-free survival (RFS) and overall survival (OS) after surgery in patients with GC and high and low BAF indices. The cutoff value of the BAF index was set at 1.522 based on ROC analysis. Statistical analyses were performed using log-rank tests.

Supplementary Figure 5

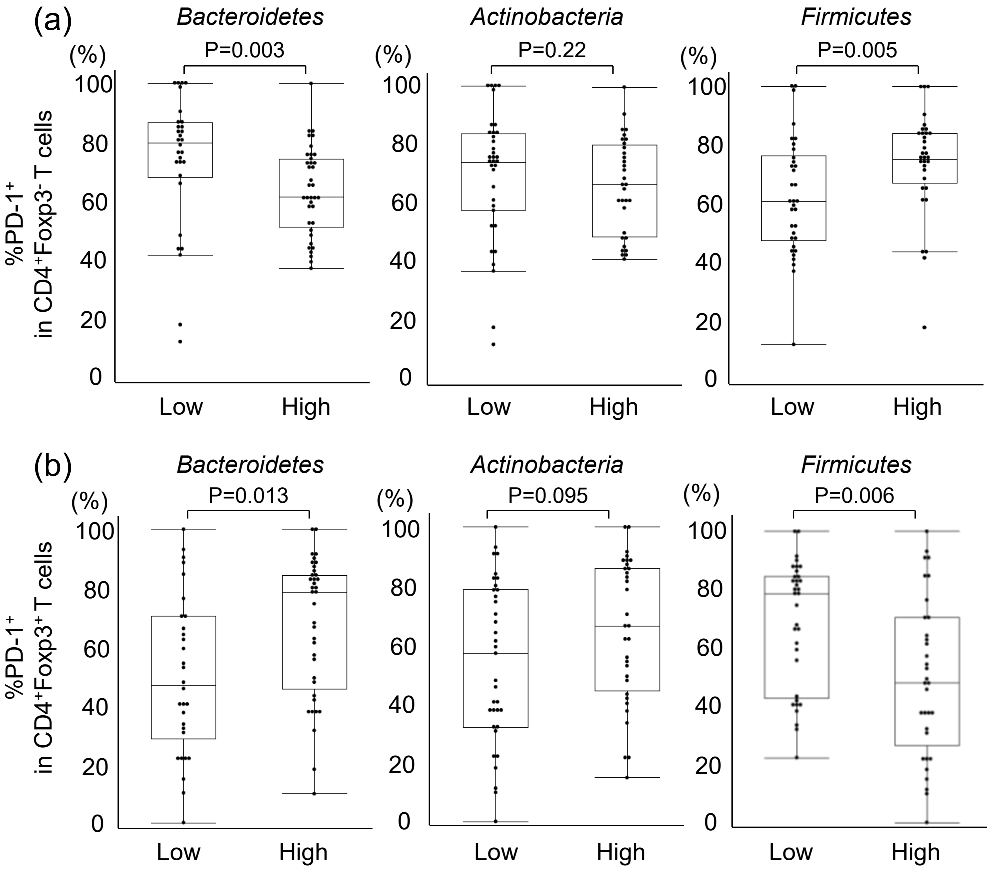

Supplementary Fig. S5 Correlation between bacteria-derived DNA (b-DNA) and tumor-infiltrating lymphocytes (TILs).

(a) The frequency of PD-1 expression in CD4+FoxP3- T cells in high and low patients with Bacteroidetes, Actinobacteria, and Firmicutes. (b) The frequency of PD-1 expression in CD4+FoxP3+ T cells in high and low patients with Bacteroidetes, Actinobacteria, and Firmicutes. Comparison between the two groups was performed using the Mann–Whitney U test.
